# Event-Related Potentials as Markers of Efficacy for Combined Working Memory Training and Transcranial Direct Current Stimulation Regimens: A Proof-of-Concept Study

**DOI:** 10.3389/fnsys.2022.837979

**Published:** 2022-04-25

**Authors:** Sara Assecondi, Bernardo Villa-Sánchez, Kim Shapiro

**Affiliations:** ^1^Center for Mind/Brain Sciences, University of Trento, Rovereto, Italy; ^2^Visual Experience Laboratory, School of Psychology, University of Birmingham, Birmingham, United Kingdom; ^3^Center for Human Brain Health, University of Birmingham, Birmingham, United Kingdom

**Keywords:** working memory training, plasticity, electroencephalography, young adults, electrophysiological markers, non-invasive brain stimulation, transcranial direct current stimulation, event related potential

## Abstract

Our brains are often under pressure to process a continuous flow of information in a short time, therefore facing a constantly increasing demand for cognitive resources. Recent studies have highlighted that a lasting improvement of cognitive functions may be achieved by exploiting plasticity, i.e., the brain’s ability to adapt to the ever-changing cognitive demands imposed by the environment. Transcranial direct current stimulation (tDCS), when combined with cognitive training, can promote plasticity, amplify training gains and their maintenance over time. The availability of low-cost wearable devices has made these approaches more feasible, albeit the effectiveness of combined training regimens is still unclear. To quantify the effectiveness of such protocols, many researchers have focused on behavioral measures such as accuracy or reaction time. These variables only return a global, non-specific picture of the underlying cognitive process. Electrophysiology instead has the finer grained resolution required to shed new light on the time course of the events underpinning processes critical to cognitive control, and if and how these processes are modulated by concurrent tDCS. To the best of our knowledge, research in this direction is still very limited. We investigate the electrophysiological correlates of combined 3-day working memory training and non-invasive brain stimulation in young adults. We focus on event-related potentials (ERPs), instead of other features such as oscillations or connectivity, because components can be measured on as little as one electrode. ERP components are, therefore, well suited for use with home devices, usually equipped with a limited number of recording channels. We consider short-, mid-, and long-latency components typically elicited by working memory tasks and assess if and how the amplitude of these components are modulated by the combined training regimen. We found no significant effects of tDCS either behaviorally or in brain activity, as measured by ERPs. We concluded that either tDCS was ineffective (because of the specific protocol or the sample under consideration, i.e., young adults) or brain-related changes, if present, were too subtle. Therefore, we suggest that other measures of brain activity may be more appropriate/sensitive to training- and/or tDCS-induced modulations, such as network connectivity, especially in young adults.

## Introduction

During a normal day we are immersed in a continuous flow of information to which we must react, understand and/or take actions. Current societal demands require us to do more things in a shorter timeframe, putting increasing demands on our cognitive resources (Dresler et al., 2019). Whereas cognitive enhancement has become the holy grail, it is important to note their underpinning cognitive training programs have their roots in plasticity, i.e., the brain’s ability to adapt to the ever-changing cognitive demands imposed by the environment. The possibility of improving memory and other cognitive functions, together with the availability of low-cost wearable devices, has fostered the development of cognitive training regimens combined with non-invasive brain stimulation (Coffman et al., 2014), albeit with somewhat contradictory results (Ke et al., 2019; Shires et al., 2020). Non-invasive brain stimulation, specifically transcranial direct current stimulation (tDCS) promotes cognitive plasticity of active networks (Stagg and Nitsche, 2011; Bikson and Rahman, 2013; Reato et al., 2019; Kronberg et al., 2020) and can now be delivered by a variety of devices, including those for home use, in healthy and clinical populations (Van de Winckel et al., 2018; Garcia-Larrea et al., 2019; Bornheim et al., 2020; Gough et al., 2020; Gulley et al., 2021).

The choice of several experimental factors plays a critical role in the efficacy of combined tDCS and cognitive training regimens, from the size and position of the electrodes, which should be congruent with the targeted network (Trumbo et al., 2016; Ruf et al., 2017) to the duration and intensity of the electrical current (Teo et al., 2011; Nikolin et al., 2018) and the duration, difficulty and scheduling of the training sessions (Berryhill, 2017; Hurley and Machado, 2018; Weller et al., 2020). Moreover, individual differences may influence the outcome of these interventions (Kosslyn et al., 2002), consistent with the notion that cognitive and electric brain stimulation, are both state-dependent techniques, i.e., their effect depends on the current substrate’s state. Individuals starting the training with limited cognitive resources respond better to stimulation than their more equipped counterpart (Tseng et al., 2012; Hsu et al., 2014; Benwell et al., 2015; Wang et al., 2018; Matysiak et al., 2019; Assecondi, 2021; Krebs et al., 2021), although opposite results have been reported (Berryhill and Jones, 2012; Jones et al., 2015).

An often-used task in working memory training regimens is the “n-back” task (Kirchner, 1958). In a typical n-back task, participants watch or listen to a stream of stimuli, presented sequentially, and respond by comparing one stimulus to those occurring “n” before for a match. Each trial of the n-back task involves several steps, from encoding, to maintenance and updating of information (Chen et al., 2015), as well as inhibitory processes (Oberauer et al., 2003) and control of interference (Kane et al., 2007). One limitation of many combined tDCS and working memory studies is the outcome measure used, which often reduces to a behavioral one, such as accuracy, reaction time and/or training gains (Ortu and Vaidya, 2016). These measures summarize a series of events that occur during several millisecond epochs, and which are often aggregated into a single number, in turn losing reference to underlying processes. In contrast, electroencephalography (EEG) can measure brain activity with millisecond time resolution, thus is an excellent tool to identify neural biomarkers (Leiser et al., 2011; Sakkalis, 2011; Al-Qazzaz et al., 2014; Mamani et al., 2017; Fraga et al., 2018), but, until now, has been used mainly in clinical populations. Moreover, EEG recordings can help to clarify the neural underpinning of combining tDCS and working memory. In the context of cognitive studies, specifically training research, event-related potentials (ERPs) have shown promise as markers of efficiency and training efficacy, although results are sparse, and, in n-back tasks, mainly focused on N2, P3 and slow waves (0–800 ms post-stimulus; Pergher et al., 2018; Vilà-Balló et al., 2018).

A visual stimulus elicits a series of components. The first components we consider are short-latency visual sensory related components: P1, N1, P2. P1 is a sensory positive deflection peaking at around 100 ms post-stimulus, at lateral occipital electrodes, with amplitude sensitive to the location of spatial attention and arousal states (Luck et al., 1993; Vogel and Luck, 2000). N1 is a negative deflection with visual subcomponents peaking between 100 and 150 ms frontally and 150–200 ms parietally and occipitally, its amplitude increasing for attended items (Hillyard et al., 1998). P2, is a positive deflection following N1, peaking between 150 and 275 ms post-stimulus at frontal and central sites, larger in response to target stimuli, when target features are simple, and further increased by frequent stimuli (Luck and Hillyard, 1994).

This short-latency components are followed by mid-latency components, related to higher cognitive processes: the N2 and P3. The N2 is a negative deflection around 200–350 ms after stimulus onset in response to repetitive stimuli. In the visual domain, if the stimuli are “deviants” (infrequent) and task-relevant, the amplitude of this component, thought to be related to stimulus-categorization processes, is larger over parietal sites (Simson et al., 1977). The N2 has also been related to stimulus awareness (Patel and Azzam, 2005) and elicited by template-mismatch to a stored expected stimulus (Sams et al., 1983; Folstein and Van Petten, 2008; Daffner et al., 2012). The P3 is a positive deflection with a peak occurring around 250–500 ms after stimulus onset (Polich and Kok, 1995; Polich, 2007). Its parietal P3b subcomponent is elicited by infrequent but “expected” targets (Verleger et al., 1994), its amplitude is sensitive to probability, i.e., infrequent stimuli, and decreases with habituation and increases with task difficulty (Watter et al., 2001; Bailey et al., 2016; Lubitz et al., 2017; Scharinger et al., 2017; Vilà-Balló et al., 2018). The P3 has also been associated with memory engagement (Azizian and Polich, 2007). Thus, stimulus encoding that promotes successful memory storage to facilitate retrieval and recognition produces increased P300-like amplitude. Moreover, this waveform is sensitive to individual differences (Dong et al., 2015), with smaller P300 amplitudes in individuals with lower working memory capacity, and it is sensitive to age (Saliasi et al., 2013; Gajewski and Falkenstein, 2014; Schapkin and Freude, 2014) with older individuals showing a smaller amplitude. The P3 is also a marker for mental workload (Brouwer et al., 2012), that is, the ratio between external cognitive demand and individual resources. P3 amplitude decreases for increasing workload, i.e., when demands are high and resources are close to maximum capacity the mental workload will be at the limit (O’Donnell and Eggemeier, 1986; Kantowitz, 2000).

Finally, late components, likely related to feedback mechanisms, have been studied in the context of working memory, and functionally different negative and positive slow waves have been identified (Rösler and Heil, 1991). The positive component is strongest at parietal sites, peaking between 500 and 1,000 ms after stimulus onset, and has been linked to explicit recognition memory with its amplitude reflecting what is known as the “old-new” effect (Friedman and Johnson, 2000), in both long- and short-term memory paradigms (Danker et al., 2008). This late component is believed to index the top-down allocation of attention in a memory recollection process (Mecklinger, 1998). In an n-back task, this component is sensitive to load and relates to active maintenance of information (Ruchkin et al., 1990, 1995; Bailey et al., 2016; Vilà-Balló et al., 2018).

Findings are scarce when the n-back task is combined with tDCS, mainly focusing on the P3 component or on neural oscillations. The P3 amplitude increases when anodal tDCS is applied offline (Keeser et al., 2011) or online (Nikolin et al., 2018). Zaehle et al. (2011) found no tDCS-related effects on the P3 component but a stimulation-dependent increase in oscillatory power (theta, alpha, and beta) when anodal stimulation was applied, whereas Hill et al. (2017) found no effects on working memory but did find an effect of increased cortical excitability.

When training is considered, i.e., repetition over multiple sessions of the same task with varying difficulty (McEvoy et al., 1998), most studies have focused on the P3 component. Cognitive training has been shown to modulate ERP components during an n-back outcome task, increasing frontal and parietal P3 amplitude (both P3a and P3b subcomponents, respectively), in young (Pergher et al., 2018), and in old adults (Gajewski and Falkenstein, 2014, 2018; Tusch et al., 2016; Chainay et al., 2021). While the P3 amplitude correlates with performance, behavioral effects are not always found (Tusch et al., 2016). Interestingly, Salmi et al. (2019) found that an adaptive dual n-back task caused a behavioral improvement in the training group, accompanied by a decreased load-effect in the P2-N2-P3 complex, but the pattern was reversed at some latencies in the control group.

To our knowledge, only one study has looked at the electrophysiological correlates of n-back memory training combined with brain stimulation (Dong et al., 2020). Dong and colleagues looked at changes in ERP during an n-back task, 1 day and 20 days after a 10-day working memory training with 2 mA tDCS over the left dorsolateral prefrontal cortex and found an overall increase in P3 amplitude 1 day after the end of training, regardless of whether participants received active or sham tDCS. Twenty days after training, however, the group receiving active tDCS showed further changes to the P3 amplitude, while the sham group did not.

With the spread of off-the-shelf stimulation devices and the proliferation of cognitive training programs, it is of paramount importance to better understand if changes in individual memory performance in response to such interventions correspond to measurable changes in brain activity. We chose to focus on ERPs as they are well suited for use with home-monitoring EEG devices, usually equipped with a limited number of recording channels. To explore the neural correlates of working memory training combined with non-invasive brain stimulation, we recorded ERPs in young adults during a spatial n-back task, at three points in time: at baseline, after 3 days of working memory training (same task as the one tested, with active or sham tDCS), and a month later. As research on the topic is scarce, our analysis is mainly exploratory. We focused on ERP components elicited by visual stimuli, such as those involved in the working memory task we used, and we addressed if and how these components are sensitive to training, tDCS, or both.

Based on the limited literature available, we chose to make predictions only in the context of the P3. Specifically, we hypothesized a modulation of the P3 in response to training, not necessarily accompanied by behavioral changes, and an effect of the stimulation, i.e., stronger at follow up.

## Materials and Methods

### Participants

Twenty-six healthy adults [age 20.5 ± 3.9, range (18,32)] were recruited through the University of Birmingham (UoB) Research Participation Scheme and adverts, receiving either study credits or monetary compensation for their participation. Individuals who did not fulfill safety criteria for tDCS (Antal et al., 2017), had cognitive training or brain stimulation in the previous 6 months, had a history or familiarity with epilepsy, or were color-blind, were excluded from participating. A power analysis, focusing on the training task, demonstrated that, for small effects ($\eta_{p}^{2}$ = 0.1) in a 2 (STIMULATION) × 3 (TIME) design, an average correlation between measurements of 0.5, a sample of 18 participants yielded a 80% power (alpha = 0.05; Faul et al., 2007, 2009). Therefore, we believe our sample was sufficient to detect effects, at least in the training task. Of all 26 participants, two did not complete the follow up session, but were still included in some aspects of the analysis, and one was excluded because of missing data. Follow up took place after about 5 weeks [5.6 ± 0.9, range (4.1, 8.3)]. The study was approved by the UoB Ethics Committee (ERN_12-1002AP18), and all participants gave written informed consent before taking part. Data analyzed in this article are part of a larger experiment, as described in the experimental procedure, but only the aspects relevant to the current study are described in detail.

### Experimental Procedure

**FIGURE 1:**
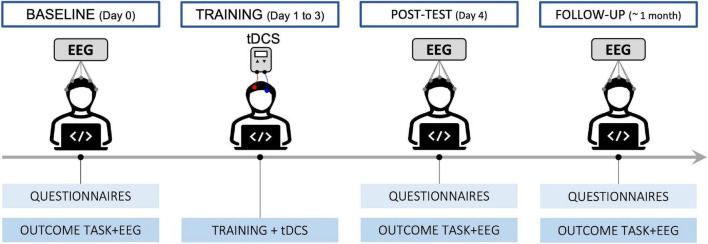
Diagram describing the timeline of the procedure.

The intervention always started on a Monday to guarantee consecutive days between the baseline and post-test. A diagram of the procedure is shown in Figure 1. At recruitment, participants were pseudo-randomly allocated to the ACTIVE or SHAM group. At day 0 (T0 – BASELINE), after filling in questionnaires on their lifestyle and cognitive state, since these variables may impact cognitive performance, they performed a change detection (CD) task, to measure their working memory capacity, followed by the EEG setup that lasted approximately 40 min. After completing a psychomotor vigilance task to assess their alertness, we recorded resting state EEG for 10 min. Participants then completed a series of computerized cognitive tests (attention network tasks and spatial and visual n-back), while their brain activity was recorded. Finally, they filled out feedback questionnaires on the cognitive tests. The procedure lasted approximately 2.5 h.

From day 1 to day 3 (T1, T2, and T3), participants took part in a training session. After reporting on their level of motivation and expectations on each day, they performed an adaptive spatial n-back task (the same as the one performed at baseline) while receiving active or sham tDCS for 21 min. They then provided feedback on any side effects of the stimulation. Including setup, each training session lasted approximately 45 min.

On day 4 (T4 – POST-TEST), participants repeated the same test battery undertaken at T0 (except for questionnaires on lifestyle, the trail making test, and habitual sleepiness). The session lasted about 2.5 h.

Between 1 month and 6 weeks later, participants returned to complete another assessment (T5 – FOLLOW UP), with the same test battery as T4. At the end they were also asked additional feedback on the intervention and on tDCS blinding. This last session lasted about 2.5 h.

### Outcome Measures

**FIGURE 2:**
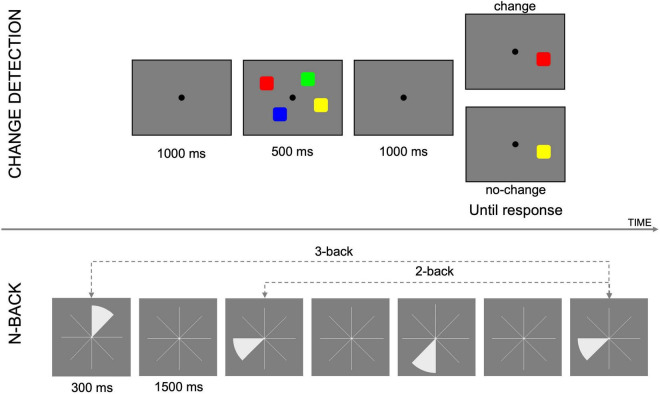
Exemplification of tasks and stimuli used in this study. Above, change detection task; below, n-back task.

Outcome measures were extracted from three different tasks: the attention network task, a visual n-back and a spatial n-back, administered using Matlab (R2017b) and the Psychophysics Toolbox extensions (Brainard, 1997; Pelli, 1997; Kleiner et al., 2007). In the following, we describe the psychomotor vigilance task, in addition to the outcome cognitive tasks analyzed in this work (i.e., CD and spatial n-back task). An example of the tasks and stimuli used is shown in Figure 2.

#### Psychomotor Vigilance Tasks

This task (Loh et al., 2004) was used to monitor participants’ alertness before starting EEG recordings. The task consisted of 50 trials of a single choice RT task. The participant fixated on a cross in the center of the screen, pressing the down arrow on the keyboard when the cross changed into a dot. The inter-stimulus interval was uniformly distributed between 2 and 10 s. If the participant pressed the arrow key within the maximum allowed response time, the dot turned green, otherwise it turned red, and accuracy and reaction time were recorded.

#### Change Detection Task

This task (Luck and Vogel, 1997) was used to obtain an individual measure of working memory capacity. Participants were shown a memory array, containing a variable number of colored squares, for 500 ms, followed by a retention interval of 1,000 ms, and a test array. The test array contained only one colored square and participants respond by pressing the key “L” (“no change”) if the color of the square in the test array was the same as the square in the same position in the memory array, or “A” (“change”), if the color was different. The number of items in the memory array varied between 4, 6, 8 (SETSIZE). Participants completed 3 blocks of 48 trials each, with half of the trials being “change,” and the other half being “no change” trials, and setsizes fully balanced. Percentage of hits (H), misses (M), correct rejection (CR), and false alarm (FA), and reaction time were recorded. D-prime (*d’*), capacity (K), and criterion (or C) were calculated, for each session (T0, T4, and T5) and setsize (4, 6, 8), as follows:


$$\begin{array}{c} d_{s,t}^{{}^{\prime }}=z\left(H_{s,t}\right)-z\left({\text{FA}}_{s,t}\right)\\ C_{s,t}=-0.5\times \left[z\left(H_{s,t}\right)+z\left({\text{FA}}_{s,t}\right)\right]\\ K_{s,t}=s\times \left(H_{s,t}-{\text{FA}}_{s,t}\right) \end{array}$$

Where *z*(.) indicates the *z*-transfom, *s* the setsize, *t* the time (T0, T4, and T5). Then *d’* and *K* were averaged over setsizes, and we calculated short-term and long-term changes, as follows:


$$\begin{array}{c} \Delta d_{\text{ST}}^{{}^{\prime }}=d_{\mathrm{T4}}^{{}^{\prime }}-d_{\mathrm{T0}}^{{}^{\prime }};\Delta d_{\text{LT}}^{{}^{\prime }}=d_{\mathrm{T5}}^{{}^{\prime }}-d_{\mathrm{T0}}^{{}^{\prime }}\\ \Delta C_{\text{ST}}=C_{\mathrm{T4}}-C_{\mathrm{T0}};\Delta C_{\text{LT}}=C_{\mathrm{T5}}-C_{\mathrm{T0}}\\ \Delta K_{\text{ST}}=K_{\mathrm{T4}}-K_{\mathrm{T0}};\Delta K_{\text{LT}}=K_{\mathrm{T5}}-K_{\mathrm{T0}}\\ \Delta {\text{RT}}_{\text{ST}}={\text{RT}}_{\mathrm{T4}}-{\text{RT}}_{\mathrm{T0}};\Delta {\text{RT}}_{\text{LT}}={\text{RT}}_{\mathrm{T5}}-{\text{RT}}_{\mathrm{T0}}, \end{array}$$

where ST and LT stand for short-term and long-term, respectively.

#### Spatial n-Back

A non-adaptive spatial n-back task was used to assess working memory performance (Kirchner, 1958). During each trial one slice of an eight-slice pie chart would turn white, for 300 ms. The slice then disappeared, leaving the empty pie chart for 1,500 ms. During this interval, participants were expected to make a judgment on the similarity between the position of the last slice and “n” slices before, pressing the key “A” for “different” or “L” for “same.” The experiment consisted of 6 blocks of 40+n trials each (33% change), for each LOAD “n” considered (*n* = 2, 3, 4). At the end of each block participants received feedback on their performance. Before performing the actual task, participants had the chance to practice for *n* = 1 and *n* = 2. Apart from *K*, the same variables as in the CD task were calculated.

### Training Task

An adaptive version of the outcome spatial n-back was used to train working memory. In this case, though, the difficulty (load “n”) of the task was reassessed at the beginning of each block, based on participant’s performance on previous block. Each training session (T1–T3) consisted of 20 blocks of 20+n trials, starting from *n* = 2 and increasing “n” if accuracy of the previous block was above 90% or decreasing if it was below 60%. The dependent variable was the average “n” $\left(\bar{n}\right)$ for a given session. To correct for differences between groups at the first day of training (T1), we calculated changes in $\bar{n}$ ($\Delta \bar{n}$) with respect to T1 as:


$$\begin{array}{c} \Delta {\bar{n}}_{\mathrm{T2}}={\bar{n}}_{\mathrm{T2}}-{\bar{n}}_{\mathrm{T1}}\\ \Delta {\bar{n}}_{\mathrm{T3}}={\bar{n}}_{\mathrm{T3}}-{\bar{n}}_{\mathrm{T1}} \end{array}$$

Where $\Delta {\bar{n}}_{\mathrm{T2}}$ and $\Delta {\bar{n}}_{\mathrm{T3}}$ are changes at the second (T2) and third session (T3) of training with respect to the first (T1).

### Transcranial Direct Current Stimulation

The non-invasive direct current stimulation (tDCS) targets the right dorsolateral prefrontal cortex (DLPFC) as it has been shown that tDCS to the DLPFC can modulate working memory in young adults (Au et al., 2016; Assecondi, 2021). A bipolar setup (Starstim system, Neuroelectrics Inc., Barcelona, Spain) included two round Ag/AgCl electrodes (area = 3.14 cm^2^) filled with conductive gel (SIGNAGEL^®^) and placed on F4 (active electrode) and Fp1 (return electrode). Impedances were kept below 10 kΩ at any time. We used a current intensity of 2 mA for 20 min, preceded by 30 s ramping up and followed by 30 s ramping down (total stimulation time = 21 min), obtaining a current density of approximately 0.6 mA/cm^2^, slightly higher than the one obtained with larger electrodes, but still well below the threshold for tissue damage (Liebetanz et al., 2009; Bikson et al., 2016; Antal et al., 2017). Sham stimulation used the same setup as in the active condition but after ramping up the current was returned to zero and the process repeated after 20 min time interval (total sham stimulation time = 21 min).

### Electroencephalography Data Acquisition and Analysis

#### Electroencephalography Recording

Electroencephalography data were recorded throughout the baseline (T0), post-test (T4), and follow up (T5) session. The recording apparatus consisted of a multi-channel ActiveTwo BioSemi (BioSemi, Amsterdam, Netherlands) amplifier and an elastic fabric electrode cap. EEG was recorded reference-free, with a Common Mode Sense active electrode and a Driven Right Leg passive electrode as ground. Ag/AgCl pin-type active electrodes, filled with conductive gel (SIGNAGEL^®^), were placed according to the 10–20 electrode systems, and sampled at 1,024 Hz. Electrode offset was kept stable at around 25 mV. Three additional flat electrodes were used on the left and right mastoids, for offline re-referencing, and below the right eye, to measure vertical electrooculogram.

#### Electroencephalography Preprocessing

Electroencephalography data were pre-processed and analyzed using EEGLAB (Delorme and Makeig, 2004) and ERPLAB (Lopez-Calderon and Luck, 2014) for MATLAB^®^ (Mathworks). Continuously recorded data, re-referenced to average mastoids at import, was filtered to 256 Hz, for subsequent downsampling at 512 Hz, and high-pass filtered at 0.16 Hz to remove slow drifts. Channels whose amplitude exceeded 150 μV for more than 20% of the recording duration or whose power spectral density was 5 dB larger than the average power over all channels, were automatically labeled as bad. Channels marked as bad were visually inspected and confirmed for rejection. To further remove remaining artifacts, the EEG was filter to 0.16 and 100 Hz and decomposed by applying ICA to good channels only. ICs components with a 90% chance of being an artifact (Muscle, Eye, Heart, Line Noise, and Channel Noise) were automatically identified and removed using ICLabel^1^ (Pion-Tonachini et al., 2019) and eyeCatch^2^ (Bigdely-Shamlo et al., 2013). The signal was then reconstructed without the artefactual components and channels marked as bad were interpolated.

#### Event-Related Potentials Extraction

The cleaned EEG was low-pass filtered (40 Hz cut-off, order 2) to further attenuate noise, before extracting epochs from 200 ms before to 1,495 ms after stimulus onset. Epochs were baseline corrected (−200 to 0 ms pre-stimulus) and those with a peak-to-peak amplitude larger than 150 μV (±75 μV) were rejected (mean percentage of rejected epochs 6.7 ± 9.7%, see Supplementary Material). We sorted the data into experimental conditions and averaged the epochs to obtain an average ERP for each participant, load (“n”), session (T0, T4, and T5), and condition (hits, missed, correct rejections, and false alarms).

#### Electroencephalography Analysis

**FIGURE 3:**
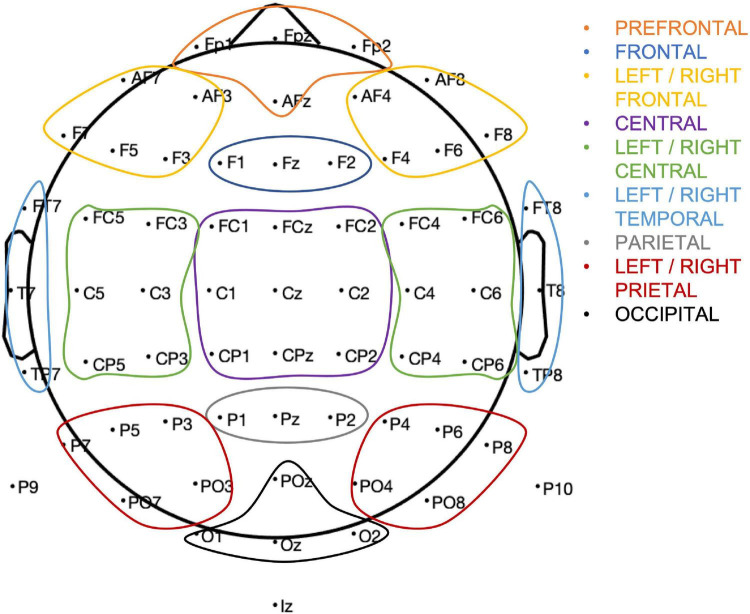
Region of interest and corresponding electrode assignment.

**FIGURE 4:**
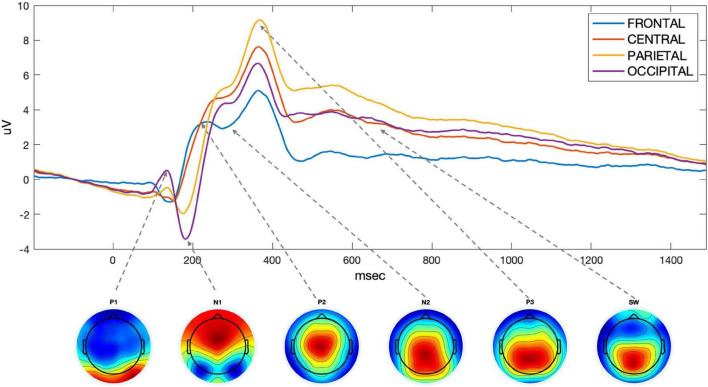
Time-course of the ROIs selected to measure ERP components. Below, topographies of the components considered, with the arrow linking them to the corresponding peak on the ROIs time course.

Thirteen region of interest (ROI) were defined based of electrode proximity and are shown in Figure 3. An ERP components’ amplitude (*A*) was measured as the average amplitude over the temporal window of interest on the average of hits and correct rejections, for each participant. As we are interested in changes in an overall measure of performance, we average hits and correct rejection. The ROI and the individual time windows were identified by visual inspection of the grand average for all participants (ACTIVE and SHAM) and Hits and Correct Rejection and based on literature (Table 1). An example of components’ topography and ERPs, averaged over all participants, loads (“n”) and conditions, is shown in Figure 4.

**TABLE 1:** Overview of the time ranges and channels used to measure ERP components.

Component	Time interval (ms)	ROI	Channels in ROI
P1	100–160	OCCIPITAL	POz,O1,O2,Oz
N1	160–210	LEFT/RIGHT PARIETAL	P4,P6,P8,P10,PO4,PO8, P3,P5,P7,P9,PO3,PO7
P2	200–280	CENTRAL	FC1,FC2,FCz,C1,C2,Cz, CP1,CP2,CPz
N2	260–340	PARIETAL	P1, P2, Pz
P3	320–420	PARIETAL	P1, P2, Pz
SW	500–1,000	PARIETAL	P1, P2, Pz

For each component, we calculated short-term and long-term amplitude changes with respect to baseline (T0), as follows:


$$\begin{array}{c} \Delta A_{\text{ST},i}=A_{\mathrm{T4},i}-A_{\mathrm{T0},i}\\ \Delta A_{\text{LT},i}=A_{\mathrm{T5},i}-A_{\mathrm{T0},i} \end{array}$$

where ST and LT correspond to short-term (post-test) and long-term (follow up), respectively, and *i* indicates the *i-th* ERP component. Similarly, we define modulation as:


$$M_{t,i}=\frac{\left(A_{t,i}\left(\mathrm{N3}\right)-A_{t,i}\left(\mathrm{N2}\right)\right)+\left(A_{t,i}\left(\mathrm{N4}\right)-A_{t,i}\left(\mathrm{N3}\right)\right)}{2};$$

where N2, N3, N4 indicates the LOAD, and *i* is the *i-th* component. Then, similarly, changes in modulation were defined, for each component, as:


$$\begin{array}{c} \Delta M_{\text{ST},i}=M_{\mathrm{T4},i}-M_{\mathrm{T0},i}\\ \Delta M_{\text{LT},i}=M_{\mathrm{T5},i}-M_{\mathrm{T0},i} \end{array}$$

### Strategy Instructions and Questionnaires

**FIGURE 5:**
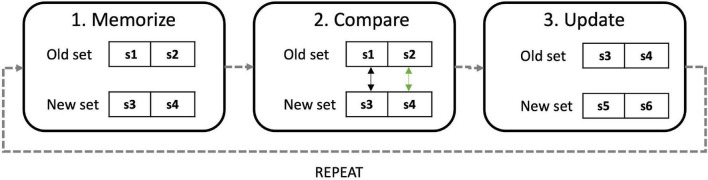
Diagram explain strategy instructions: participants were instructed to create a memory array with the first “n” items in the stream, then to create a second array with the next “n” items. At this point they could compare “new” items with “old” ones and respond. Finally, they had to discard the “old” array, not necessary, and create a new one. The process was repeated until the end of the stream.

Before starting the first n-back (at baseline, T0) participants were provided with instructions on how to perform the task, depicted in Figure 5. Instructions follow a general “memorize,” “compare,” and “update” strategy (Laine et al., 2018; Assecondi, 2021), applicable to n-back tasks, as we found that efficient strategy use can modulate tDCS effectiveness and can reduce noise due to interindividual differences (Lövdén et al., 2012).

To control for individual differences that could influence the training outcome, such as participants’ lifestyle and attitude toward the training, we administered a series of questionnaires listed in Table 2.

**TABLE 2:** Description of the questionnaires administered to participants.

		Questionnaire	Measured variable	Day
Individual lifestyle	Health history*	A series of questions about past health history and current medications	1
	Quality of life (QoL; WHO, 2012)	A short version of the WHOQOL-100 which assesses the well-being in four domains: physical, psychological, social, and environment	1
	Simple physical activity questionnaire (SIMPAQ; Rosenbaum and Ward, 2016)	A short questionnaire to measure the level of physical activity	1
	Familiarity with technology*	A measure of an individual’s level of experience on everyday modern technology	1
	Pittsburgh sleep quality index (PSQI; Buysse et al., 1989)	A questionnaire to record the sleep quality and disturbances	1

Cognitive state	Instantaneous	Motivation and expectation*	Feelings and attitude toward the intervention	1 to 6
		Karolinska Sleepiness Scale (Akerstedt and Gillberg, 1990)	Subjective sleepiness	1 to 6

		PANAS (Crawford and Henry, 2004)	Positive and negative attitude	1 to 6
	Habitual	Hospital anxiety and depression scale (HADS; Zigmond and Snaith, 1983)	A questionnaire to detect states of anxiety and depression	1
	Epworth sleepiness scale (Johns, 1991)	A measure of individual’s habitual sleepiness	1
	Trail making test (Reitan, 1958)	A neuropsychological test to evaluate executive abilities (i.e., mental flexibility, visual attention)	1

Feedback	Task load index (Hart and Staveland, 1988)	Task-related perceived workload	1 to 6
	Side effects of brain stimulation	Perceived side effects of tDCS	2 to 4
	Strategy feedback*	Use of strategy during tasks	6
	Blinding	Perceived experimental group	6

*Developed in-house.

The Positive Affect/Negative Affect Schedule (PANAS; Crawford and Henry, 2004) was administered at the beginning of each session (time reference: “now”). In addition, we added five additional questions on a Likert scale (1–5, on alertness, motivation, sadness, and expectation) based on one’s subjective “feeling” as mood (Osaka et al., 2013; Rączy and Orzechowski, 2021), motivation (Krawczyk and D’Esposito, 2013; Sanada et al., 2013), and expectation (Bollinger et al., 2010; Zhao et al., 2019) could modulate working memory performance. Alertness was monitored with the PVT before each EEG session, and with the KSS before each training session. Additionally, we measured perceived workload with the NASA-TLX questionnaire (Hart and Staveland, 1988), with three subscales (mental, physical, and effort) at the end of each single session. Finally, after each training session, participants reported on side effects of the stimulation and, at the end of the last session (T5), we asked them about the strategy used during training, if any, and to report to which group (ACTIVE or SHAM) they thought they had been assigned to.

### Statistical Analysis

All analysis were performed using JASP (JASP Team, 2020). We found a significant difference in working memory at baseline between ACTIVE and SHAM group, however, this is not an issue (Harvey, 2018) if appropriately considered in the analysis. Thus, when necessary, dependent variables were transformed as the difference between measurements at post-test (T4) or follow up (T5) and baseline (T0). Mean differences between groups were analyzed with either independent t-test (STIMULATION), or repeated-measure analysis of variance when time, load or setsize were considered (TIME × STIMULATION or TIME × STIMULATION × LOAD). Greenhouse-Geisser correction was applied for violation of Sphericity, as well as Holm correction for multiple comparisons in *post-hoc* test, when necessary. We reported $\eta_{p}^{2}$ and Hedges’ *g* for small sample sizes as effects size for ANOVA and *t*-test, respectively. Fisher’s exact test for small samples was used to find associations between variables. All transformed variables (changes) were also tested against zero (one-sample *t*-test). Relations between variables were assessed using Spearman’s correlation (*r*_*s*_). When considering variables with dependent observations, repeated measures correlations were used (Bakdash and Marusich, 2017). In pilot studies, aim at providing preliminary evidence of efficacy of an intervention, it is acceptable to increase the level of significance for hypothesis testing, even up to 0.25 (Schoenfeld, 1980). We decided to discuss any result with a *p* < 0.1 (Lee et al., 2014). As we report actual *p*-values for all the analysis, it is straightforward for the reader to interpret our findings in the context of a conventional alpha (0.05). As two participants did not return for follow up, short-term effects (POST-TEST) and long-term effects (FOLLOW UP) were analyzed separately to fully exploit the data available. Details of each analysis are provided in the results section before describing the corresponding results.

## Results

### Demographic and Baseline Differences

There was no difference between groups in age [*t*_(24)_ = 1.331, *p* = 0.198, *H*_*g*_ = 0.51], gender (Fisher’s exact *p* = 0.411), handedness (Fisher’s exact *p* = 1.000), or education (Fisher’s exact *p* = 0.645). Demographic characteristics were balanced between groups (see Supplementary Material). We did find, however, a difference in performance between groups at baseline [ACTIVE: 1.52 ± 0.13; SHAM: 1.99 ± 0.14; *t*_(24)_ = 2.394, *p* = 0.025, *H*_*g*_ = 0.91] but not in capacity. No significant association between experimental and perceived group assignment (ACTIVE vs SHAM stimulation) was found (Fisher’s exact test *p* = 0.594). We found no difference between groups in term of cognitive state and lifestyle (see Supplementary Table 3). We only found a significant difference in overall physical activity (as measured via the SIMPAQ) with the SHAM group being slightly more active than the ACTIVE group.

### Working Memory Performance

#### Training Gains

**FIGURE 6:**
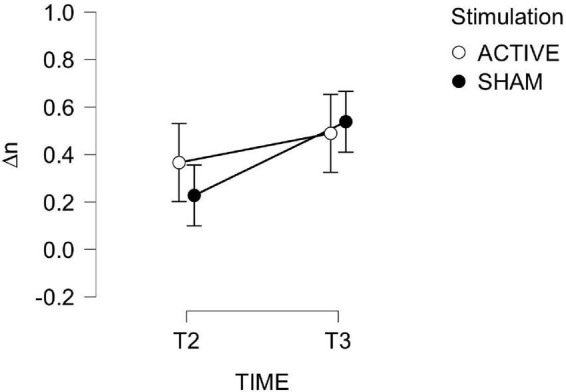
Changes in average N in the ASNBACK task (training) as a function of TIME and STIMULATION.

Training performance is depicted Figure 6. A 2-way mixed ANOVA (TIME × STIMULATION) of changes in average N in day 2 (T2) and 3 (T3) with respect to T1 $\left(\Delta {\bar{n}}_{\mathrm{T2}},\Delta {\bar{n}}_{\mathrm{T3}}\right)$ revealed no effect of TIME or STIMULATION. Improvement from baseline were significantly larger than zero only at day 3 for the ACTIVE group [ACTIVE: *t*_(12)_ = 2.572, *p* = 0.024; SHAM: *t*_(11)_ = 1.407, *p* = 0.187]. To summarize, the training regimen induced an overall improvement in (${\bar{n}}_{\mathrm{T3}}$), regardless of the stimulation, but 3 days of training were needed for this improvement to be significant.

#### Change Detection

**FIGURE 7:**
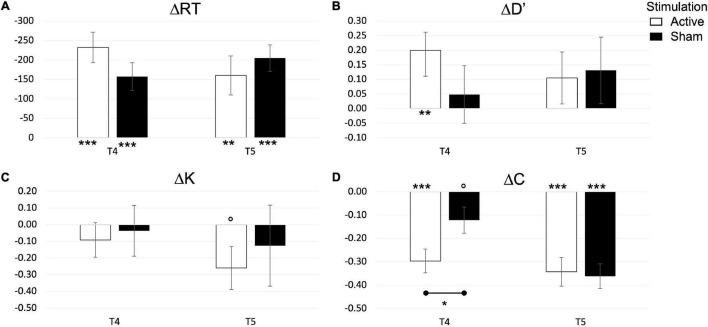
Changes in reaction time **(A)**, performance **(B)**, capacity **(C)**, and bias **(D)** in the Change detection task. *P*-values are indicated as follows: °*p* < 0.1, *p < 0.05, **p < 0.01, and ***p < 0.001.

Change detection’s behavioral performance is depicted Figure 7, while tabulated statistics is reported in the Supplementary Material. An independent *t*-test on ΔRT,Δ*d*′,Δ*C*,Δ*K*, did not reveal an effect of stimulation at post-test (T4) or follow up (T5). We further explore changes in dependent variables with a one-sample *t*-test. RT decreased in response to training and tDCS in ACTIVE [*t*_(12)_ = 5.907, *p* < 0.001] and SHAM [*t*_(11)_ = 4.415, *p* = 0.001], a reduction that remained significant at follow up [ACTIVE: *t*_(12)_ = 3.186, *p* = 0.008; SHAM: *t*_(9)_ = 5.974, *p* < 0.001]. *d’* changed significantly only in the ACTIVE group at post-test (T4) [*t*_(12)_ = 3.210, *p* = 0.007], although an effect of STIMULATION could not be detected. Changes in bias (Δ*C*) were also significant in both groups at post-test [ACTIVE: *t*_(12)_ = 5.769, *p* < 0.001; SHAM: *t*_(11)_ = 2.182, *p* = 0.052] and follow up [ACTIVE: *t*_(12)_ = 5.491, *p* < 0.001; SHAM: *t*_(9)_ = 6.863, *p* < 0.001], although the effect of STIMULATION was significant at post-test only, with *C* decreasing more in the ACTIVE than in the SHAM group [*t*_(23)_ = 2.310, *p* = 0.030]. Changes in capacity (Δ*K*) were trending toward significance in the ACTIVE group at follow up only [*t*_(12)_ = 2.009, *p* = 0.068]. To summarize, the combined cognitive training and tDCS affected the CD tasks both in a reduction in RT, surviving at follow up, and a decrease of bias (C) in the ACTIVE group. This behavior was also more evident in the ACTIVE than in the SHAM group.

#### Spatial n-Back

### Behavioral Performance

**FIGURE 8:**
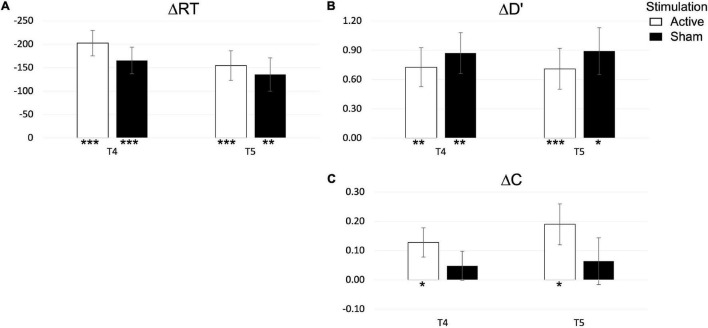
Changes in reaction time **(A)**, performance **(B)** and bias **(C)** at post-test (T4) and follow up (T5) in the SNBACK task. *P*-values are indicated as follows: *p < 0.05, **p < 0.01, and ***p < 0.001.

Behavioral performance in the SNBACK task is depicted in Figure 8, while tabulated statistics is reported in the Supplementary Material. Again, an independent *t*-test of ΔRT,Δ*d*′,Δ*C* between ACTIVE and SHAM found no main effect of STIMULATION in any of the dependent variables at post-test (T4) or follow up (T5). We further explore changes in dependent variables with a one-sample *t*-test. We find that both ΔRT and Δ*d*′ were significantly different from zero in both stimulation groups at post-test [ΔRT – ACTIVE: *t*_(12)_ = 6.855, *p* < 0.001; SHAM: *t*_(11)_ = 6.572, *p* < 0.001; Δ*d*′ – ACTIVE: *t*_(12)_ = 4.044, *p* = 0.002; SHAM: *t*_(11)_ = 3.742, *p* = 0.003] and at follow up [ΔRT – ACTIVE: *t*_(12)_ = 5.586; *p* < 0.001; SHAM: *t*_(9)_ = 3.338, *p* = 0.009; Δ*d*′ – ACTIVE: *t*_(12)_ = 4.953, *p* < 0.001; *t*_(9)_ = 2.888, *p* = 0.018]. On the other hand, Δ*C* was only significant in the ACTIVE group at post-test [*t*_(12)_ = 2.329, *p* = 0.038] and at follow up [*t*_(12)_ = 2.511, *p* = 0.027]. To summarize, the regimen had an overall effect with and reduction of RT and an increase in *d*′, which were maintained at follow up, and an increase in *C*, with changes remaining at follow up, only in the ACTIVE group.

### Event Related Potential

The potential of ERP to detect training-related and stimulation-related change was investigated, in two aspects: (1) the correlation of amplitude with task performance; (2) intervention-related effects on ERP components: that is, changes in amplitude and load-related modulation from baseline to follow up.

#### Association Between Behavior and Event-Related Potentials

To understand the function of directional changes in ERP amplitude, we computed the correlation between peak amplitude and performance (*d’*), in the SNBACK task, for all levels of LOAD at baseline (T0). Correlations are summarized in Table 3. Although all correlations show only small to moderate effects, we found that N1 negatively correlates with performance, while N2, P3, SW correlate positively with *d’* at baseline (T0).

**TABLE 3:** Correlation between amplitude of ERP components and performance (*d’* from SNBACK task) at baseline (T0).

RM-correlation ERP amplitude <-> performance (*d’*)					
ERP component	df	*r*	*p*	CI_low	CI_high
P1	51	−0.07	0.614	−0.34	0.21
N1	51	−0.23	0.097	−0.48	0.05
P2	51	0.00	0.997	−0.28	0.28
N2	51	0.42	0.002	0.16	0.62
P3	51	0.48	<0.001	0.24	0.67
SW	51	0.57	<0.001	0.35	0.73

*ACTIVE and SHAM participants are grouped together, and correlations are calculated by aggregating points from “n” levels, but taking into account the dependency in the data.*

#### Intervention-Related Effects on Event-Related Potentials Components

**FIGURE 9:**
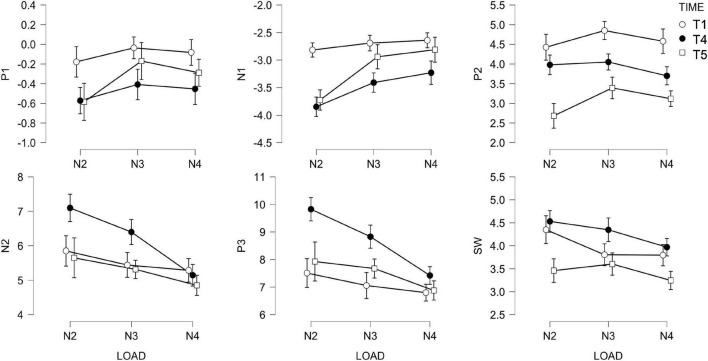
Amplitude of ERP for each LOAD (*n* = 2, *n* = 3, and *n* = 4), TIME (T0, T4, and T5) and component.

**FIGURE 10:**
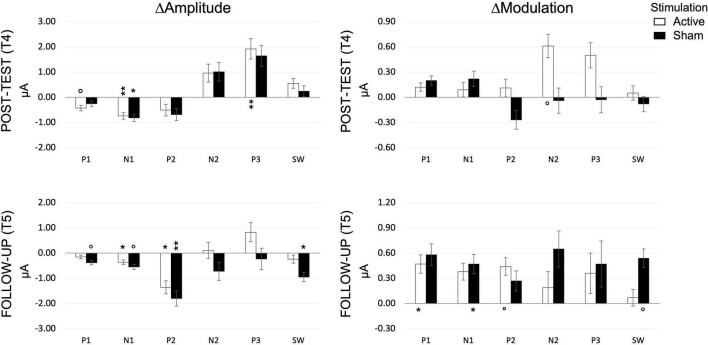
Amplitude and modulation changes at post-test (T4) and follow-up (T5), for each component, as measured during the SNBACK task. *P*-values are indicated as follows: °*p* < 0.1, *p < 0.05, **p < 0.01.

Because of the small sample size, to exploit all data available, we analyzed post-test (T4) and follow up (T5) sessions separately. Figure 9 shows the ERP amplitude for each LOAD, TIME, and component. Tabulated values are reported in the Supplementary Material. Two independent *t*-tests of amplitude and modulation changes (Δ*A*_ST,*i*_;Δ*A*_LT,*i*_;Δ*M*_ST,*i*_;Δ*M*_LT,*i*_) between ACTIVE and SHAM, at T4 and T5, revealed no effect of STIMULATION. We further tested short- (T4) and long-term (T5) changes in amplitude and modulation against zero, within the ACTIVE and the SHAM groups, separately. Changes in amplitude and modulation for each component are depicted in Figure 10.

##### P1 Component

*A*_*P*1_ did not correlate with performance. Changes in *A*_*P*1_ (Δ*A*_*P*1_) were not consistent between STIMULATION groups, with only a significant decrease in the ACTIVE at post-test (T4) [*t*_(12)_ = 1.858, *p* = 0.088] and a significant increase in the SHAM at follow up (T5) [*t*_(9)_ = 1.846, *p* = 0.098]. P1 amplitude modulation (Δ*M*_*P*1_ differentiation between loads) increased in ACTIVE only at follow up [*t*_(12)_ = 2.818, *p* = 0.016].

##### N1 Component

N1 responded to training in both groups by decreasing in amplitude [ACTIVE: *t*_(12)_ = 3.706, *p* = 0.003; SHAM: *t*_(11)_ = 2.475, *p* = 0.031], a change, corresponding to a larger N1, that remained at follow up [ACTIVE: *t*_(12)_ = 2.501, *p* = 0.028; SHAM: *t*_(9)_ = 2.187, *p* = 0.057]. N1 modulation (Δ*M*_*N*1_) increased at follow up, reflecting a better differentiation between loads, only in the SHAM group [*t*_(9)_ = 2.417, *p* = 0.039] but no effects were seen at post-test.

##### P2 Component

P2 amplitude change (Δ*A*_*P*2_) was not sensitive to training at post-test (T4) while it decreased significantly at follow up (T5) in both ACTIVE [*t*_(12)_ = 2.224, *p* = 0.046] and SHAM [*t*_(9)_ = 4.236, *p* = 0.002]. Its modulation (Δ*M*_*P*2_) increased at follow up, only in the ACTIVE group [*t*_(12)_ = 2.085, *p* = 0.059].

##### N2 Component

N2 amplitude did not change significantly either at post-test or at follow up. Only the ACTIVE group showed a significant increase in N2 modulation at post-test [*t*_(12)_ = 1.877, *p* = 0.085], which did not survive at follow up.

##### P3 Component

P3 amplitude increased (Δ*A*_*P*3_ > 0) at post-test (T4) in the ACTIVE group only [*t*_(12)_ = 3.255, *p* = 0.007], but no significant changes at follow up or in modulation could be detected.

##### SW Component

SW amplitude decreased only in SHAM at follow up [*t*_(9)_ = 2.756, *p* = 0.022], and its modulation increased only in SHAM at follow up [*t*_(9)_ = 2.143, *p* = 0.061].

### Participants’ Feedback

Not all participants reported to have used the strategy instructed, but all but one used “a” strategy (tabulated data are reported in the Supplementary Material). We found no significant association between STIMULATION groups and those who used the strategy instructions given during the training [*X*^2^_(1, *N* = 25)_ = 0.962, *p* = 0.327], or a different strategy [*X*^2^_(1, *N* = 25)_ = 1.128, *p* = 0.288]. There was also no difference in perceived efficacy between ACTIVE and SHAM. Perceived efficacy correlated significantly (Pearson’s *r* = 0.485, *p* = 0.014, *N* = 25) with training gains.

We also found an effect of stimulation on mental demand and effort, but only with ACTIVE higher than SHAM. This effect, though, reflects baseline differences between groups, as those with lower working memory performance at baseline, also showed more mental demand and effort.

## Discussion

In the present study we investigated electrophysiological correlates of working memory training combined with non-invasive brain stimulation (tDCS). Young adults completed a 3-day regimen while their behavioral performance was monitored. Before the training, participants completed a non-adaptive version of the same task used for training, while their brain activity was recorded. In addition, individuals were asked to complete a CD task to obtain a measure of their working memory capacity.

We were interested in the relation between the amplitude of ERP components and behavioral performance, in response to combined working memory training and tDCS. To the best of our knowledge only few studies have investigated the effect of combined training and tDCS regimens on brain activity. Jones and co-authors (Jones et al., 2020) found that active tDCS during a short working memory training (a CD task over 4 days) improved theta-gamma interactions in the fronto-parietal network. Dong et al. (2020) used a high-definition tDCS setup during a verbal and spatial n-back training for 10 days, and found a modulation of P3 between ACTIVE and SHAM 20 days after training. There is a widespread use of home devices for improving cognitive functions, although claims of effectiveness often lack backing by thorough scientific evaluation. Moreover, gamers are using more and more unregulated brain stimulation devices to improve their performance (Santarnecchi et al., 2013; Wurzman et al., 2016). We decided to follow the ERP approach (Dong et al., 2020), as it is more suitable for use with home devices where usually only a limited number of EEG channels are available. Given the lack of literature on the topic, we did not have a clear a-priori hypothesis. Therefore, we decided to take an exploratory approach and evaluate if and how ERP components’ absolute amplitude and amplitude modulation, i.e., amplitude differentiation between loads, are affected by combined regimens.

### Behavioral Performance

We found an overall improvement during training, although not modulated by the stimulation. Individuals needed 3 days of training for these improvements to become significant. It is worth reminding that there are two types of variability at play in this dataset. The two groups showed a significant difference in cognitive performance at baseline, and, although we addressed the issue by using increment scores, this difference may still impact training-related improvement. Additionally, individuals with higher cognitive performance may be able to devise better strategies more rapidly than those with less capacity. We control for differences in strategy by providing strategy instructions, as initial strategy instructions reduced inter-subject variability in memory performance (Lövdén et al., 2012). When the brain’s activity is already optimized to undertake a given task, e.g., when strategy instructions are given, any additional improvement, due to further training, should be related to brain plasticity. In this case, a magnification of behavior is expected, that is, people with higher baseline capacity (which reflects past plasticity) should improve more. In our case, we would therefore expect the sham group to outperform the active: however, this is not the case as improvements are not different between conditions. This could be viewed as an effect of stimulation in the active group in support of the plasticity hypothesis. According to the magnification-compensation theory, individuals who are already using their resources optimally have less room for improvement, therefore will improve less, expecting a negative correlation between initial performance and gains from cognitive training (Lövdén et al., 2012). Finally, considering our previous findings (Assecondi, 2021), a failure to find an effect of stimulation could also be ascribed to the impossibility, given the small sample size, to focus on individuals with low working memory capacity.

In behavioral outcome variables, aside from a general unsurprising effect on RT, we found an effect of stimulation on bias (C). Specifically, in the CD task, stimulation modulated changes in C at post-test (T4) with negative changes larger in the active than in the sham group. This means that the criterion became more negative after training, which in the CD task, means that individuals were more likely to say that the color has changed even when it has not. In the SNBACK (trained) task, we did not find a main effect of stimulation, but we did find that changes were positive and only significantly larger than zero in the ACTIVE group, both at post-test and at follow up. A positive change means that the criterion became more positive, which in the SNBACK task, means that individuals were more likely to say that it was not a target, that is, it was different from the “template” they had in mind. Changes in bias were, therefore, of opposite sign in the SNBACK and in the CD, but both resulting in the individuals being more likely to say that the stimulus was different from their “memorized” template. This could be due to a change in strategy, but we found no significant association between strategy use and stimulation groups, or in attitude, and we did find a significant effect of session on positive attitude (as measured by the PANAS) but, still, not modulated by stimulation.

### Event-Related Components

We investigated the effects of the combined regimen on short-latency sensory components (P1, N1, P2), and mid-latency (N2, P3) and long-latency (SW) cognitive components. We focused on amplitude and its modulation in response to load. Overall, in an n-back task, as difficulty (“n”) goes up, so does the demand for sustained attention. These increased demands reflect a general cognitive response to increasing difficulty (McEvoy et al., 1998).

#### Short-Latency Visual Sensory Components (P1-N1-P2)

These sensory components are sensitive to low-level features of the stimuli and are modulated by attention. N1 is also larger in discrimination relative to detection tasks. N1 decreased significantly in both ACTIVE and SHAM at post-test (which is associated with better performance). Overall, N1 indicates an increased efficiency in task performance, regardless of stimulation, showing as a larger modulation of the amplitude across loads but only at follow up, only for the sham group, indicating some changes may be long lasting or need time to consolidate.

#### Mid-Latency Components (N2-P3)

The N2 and P3 components are thought to reflect active cognitive processes. Specifically, the N2 is associated with stimulus categorization and response to infrequent stimuli, while the P3 is linked to resource allocation and decision-making. We found the N2 and P3 positively correlate with performance [smaller more positive N2, and larger P3 correspond to better performance (Nikolin et al., 2018)], with the component becoming smaller with increasing load (difficulty; Kahneman, 1973; McEvoy et al., 1998; Folstein and Van Petten, 2008; Daffner et al., 2012). In response to combined training, significant changes in amplitude were detected only at post-test for P3 and only in the ACTIVE group, with P3 increasing after training. Regarding the N2, this component is sensitive to template mismatch, therefore it is possible that measuring its amplitude on the average of both targets and foils would confound the effect. An alternative marker could be the difference in amplitude between targets and foils, and how this is modulated by training interventions. However, the n-back design considers only thirty percent of trials to be targets, which leave us with too few trials for ERP analysis. We therefore decided to focus on an overall indicator of performance as the average of all correct answers. When we consider the P3 component, an increased amplitude is associated with successful memory storage (Azizian and Polich, 2007), which in turn is associated with better performance. We found this effect in the active group only, and in line with previous training studies (Gajewski and Falkenstein, 2014; Tusch et al., 2016; Pergher et al., 2018; Chainay et al., 2021). A larger P3 could also be associated with a reduction in workload, although it is worth mentioning that we did not find an effect of TIME or STIMULATION on workload, as measured by the NASA-TLX.

Finally, the P3 has been considered as a marker of motivation, or more specifically, the participant’s engagement in the task (Kleih et al., 2010; Giustiniani et al., 2020). Interestingly, Locke and Braver (2008) defined motivation as a cognitive mechanism that modulates other cognitive functions, e.g., working memory. A few studies have shown that motivation can modulate working memory (e.g., Krawczyk and D’Esposito, 2013; Sanada et al., 2013). Due to this, we considered to monitor level of motivation and expectation throughout the experiment. Although we found no effect of session or stimulation on motivation (or expectation), future work should consider addressing these potential confounds more thoroughly.

#### Long-Latency Components

The SW component is linked to explicit recognition (Friedman and Johnson, 2000) and to top-down allocation of attention during recollection. In n-back tasks the SW has been linked to active maintenance of information (Gevins et al., 1996). Contrary to previous studies, we found that SW was not modulated by task difficulty (Daffner et al., 2012; Bailey et al., 2016) either before or after training, although its amplitude correlated with task performance.

### Limitations, Conclusions, and Future Directions

Some limitations to our study are worth discussing. First and most importantly, we acknowledge baseline differences, despite a pseudo-random recruitment procedure: while this does not necessarily lead to problems (Harvey, 2018), it may lead to misleading results if these differences are not dealt with appropriately, and does limit the conclusions we can draw. Specifically, we analyzed changes in the dependent variables with respect to baseline, to account for group differences. Furthermore, we tested the changes of the dependent variables against zero to distinguish actual improvements from noise, within each stimulation group. These steps, however, only takes into account the effect of differences at baseline but not how these differences may interact with the treatment (Lövdén et al., 2012).

Second, the small sample size limits the strength of the conclusions, especially of null effects. Therefore, we refrained from overinterpreting null results. The conclusion that ERP are not sensitive to short combined training intervention still holds, the reason being either that the effects are too small or that there is not effect. Either way, our study suggest that ERP may not be the optimal marker for training efficacy in short training paradigm in portable devices.

Third, we do not have a passive control group: thus, any TIME effect cannot be attributed unequivocally to training and could be the result of practice. However, our results are in line with previous studies, and it is reasonable to assume that these are indeed training effects.

Fourth, to further complicate the picture, the pattern of responses to training intervention is also modulated by age (Shaw and Hosseini, 2021), as are the processes underlying working memory performance described below (Gajewski et al., 2018). The recruitment of young adults, and the short duration of the training protocol, could have led to a sample insensitive to the training regimen, as indicated by the weak behavioral effects. It would therefore not be surprising that brain activity also showed only small effects.

Finally, we acknowledge that brain markers other than ERPs could have been chosen to index the effectiveness of our combined training regimen. Our choice was based on the ease with which ERP components can be measured with less expensive devices, where only a limited number of recording channels are available, as well as the lack of relevant literature on the topic. We found that ERPs were not modulated, in our sample and with our study parameters, by tDCS. We found no significant effects of tDCS either behaviorally or in brain activity, as measured by ERPs. We concluded that either tDCS was ineffective (because of the specific protocol or the sample under consideration) or brain-related changes, if present, were too subtle. We suggest that other measures of brain activity, such as network connectivity, may be more appropriate/sensitive to training- and/or tDCS-induced modulations, especially in young adults.

## Data Availability Statement

The raw data supporting the conclusions of this article will be made available by the authors, without undue reservation.

## Ethics Statement

The studies involving human participants were reviewed and approved by University of Birmingham (UoB) Ethics Committee (ERN_12-1002AP18). The patients/participants provided their written informed consent to participate in this study.

## Author Contributions

SA: conceptualization, methodology, validation, data collection and curation, formal analysis, investigation, writing—original draft, writing—review and editing, and funding acquisition. BV-S: data curation, formal analysis, writing—original draft, and writing—review and editing. KS: conceptualization, methodology, resources, supervision, writing—review and editing, and funding acquisition. All authors contributed to the article and approved the submitted version.

## Conflict of Interest

A patent application has been submitted by the University of Birmingham (United Kingdom) and Dalhousie University (Canada), with SA, KS figuring among the inventors. The remaining author declares that the research was conducted in the absence of any commercial or financial relationships that could be construed as a potential conflict of interest.

## Publisher’s Note

All claims expressed in this article are solely those of the authors and do not necessarily represent those of their affiliated organizations, or those of the publisher, the editors and the reviewers. Any product that may be evaluated in this article, or claim that may be made by its manufacturer, is not guaranteed or endorsed by the publisher.

## Footnotes

1
https://github.com/sccn/ICLabel

2
https://github.com/bigdelys/eye-catch
