# Supplementary material for: Event-Related Potentials as Markers of Efficacy for Combined Working Memory Training and Transcranial Direct Current Stimulation Regimens: A Proof-of-Concept Study
Source: Front Syst Neurosci. 2022 Apr 25;16:837979. doi: 10.3389/fnsys.2022.837979 (PMC9083230; doi:10.3389/fnsys.2022.837979)
Supplement: Supplementary file 1 [file Data_Sheet_1.docx]

Supplementary Material

[1 Demographic and baseline differences 2](#_Toc90323097)

[1.1 Overall group differences 2](#_Toc90323098)

[1.2 Group differences in ERPs’ amplitude and modulation 3](#_Toc90323099)

[1.3 Baseline differences in cognitive state and lifestyle 4](#_Toc90323100)

[2 Cognitive state during training 5](#_Toc90323101)

[3 Strategy and task difficulty 6](#_Toc90323102)

[4 Possible adverse effects of brain stimulation 7](#_Toc90323103)

[5 Outcome tasks 8](#_Toc90323104)

[5.1 Change detection task 8](#_Toc90323105)

[5.2 SNBACK task 9](#_Toc90323106)

# Demographic and baseline differences

## Overall group differences

Statistical tests showed that the two groups did not differ in age, and Fisher’s exact p-valued showed no association between STIMULATION groups and gender (p=0.411), handedness (p=1.000), or education (secondary or tertiary, p =0.645).

Table S1. Demographic characteristics and descriptive statistics of the overall sample, divided by groups. For each group, we report the count N and the average score, together with its standard error, the t Welch’s statistics, corresponding p-value, and effect size (Hedge’s g). k is the working memory capacity calculated in the change detection task at baseline (T0), d’ is the performance measure calculated in the SNBACK task at baseline (T0).

| OVERALL | | ACTIVE | SHAM | t(df = 24) | p | H_g_ |
| --- | --- | --- | --- | --- | --- | --- |
| DEMOGRAPHICS | N | 13 | 13 | -- | -- | -- |
|  | Age | 21.54 ± 1.25 | 19.54 ± 0.83 | 1.331 | 0.198 | 0.51 |
|  | Gender (F/M) | 7/6 | 8/3 | -- | -- | -- |
|  | Handedness (L/R) | 2/11 | 3/10 | -- | -- | -- |
| BASELINE SCORES | k in CD | 2.06 ± 0.18 | 2.64 ± 0.35 | 1.475 | 0.157 | 0.56 |
|  | d’ in SNBACK* | 1.52 ± 0.13 | 1.99 ± 0.14 | 2.394 | 0.025 | 0.91 |

##

## Group differences in ERPs’ amplitude and modulation

Table S2. Baseline (T0) differences in ERP amplitude and modulation between ACTIVE and SHAM group. For each ERP, the mean amplitude (and modulation) in uV is reported, together with the corresponding standard error (se), the t Welch’s statistics, corresponding p-value, and effect size (Hedge’s g). Significant group differences (p < 0.1) are marked in red.

| AMPLITUDE A_T0_ | | | | | | | |
| --- | --- | --- | --- | --- | --- | --- | --- |
| ERP | ACTIVE (n=13) | | SHAM (n=12) | |  |  |  |
|  | mean | se | mean | se | t (df = 23) | p | H_g_ |
| P1 | -0.211 | 0.349 | 0.259 | 0.364 | 0.932 | 0.361 | 0.361 |
| N1 | -2.706 | 0.527 | -2.615 | 0.572 | 0.117 | 0.908 | 0.045 |
| P2 | 4.298 | 0.717 | 4.902 | 0.597 | 0.641 | 0.528 | 0.248 |
| N2* | 4.716 | 0.724 | 7.089 | 0.83 | 2.163 | 0.041 | 0.837 |
| P3* | 5.895 | 0.862 | 9.246 | 0.832 | 2.787 | 0.010 | 1.079 |
| SW* | 3.679 | 0.357 | 4.556 | 0.256 | 1.967 | 0.061 | 0.761 |
| MODULATION M_TO_ | | | | | | | |
| ERP | ACTIVE (n=13) | | SHAM (n=12) | |  |  |  |
|  | mean | se | mean | se | t (df = 23) | p | Hg |
| P1 | 0.116 | 0.124 | 0.123 | 0.107 | 0.043 | 0.996 | 0.017 |
| N1 | 0.233 | 0.187 | -0.177 | 0.191 | 1.532 | 0.139 | 0.593 |
| P2 | 0.427 | 0.236 | 0.066 | 0.293 | 0.965 | 0.345 | 0.374 |
| N2 | -0.209 | 0.281 | 0.118 | 0.383 | 0.696 | 0.493 | 0.270 |
| P3 | -0.233 | 0.343 | 0.087 | 0.543 | 0.506 | 0.618 | 0.196 |
| SW | -0.152 | 0.267 | -0.57 | 0.231 | 1.174 | 0.252 | 0.455 |

## Baseline differences in cognitive state and lifestyle

Table S3. Scores differences between ACTIVE and SHAM group, for each administered questionnaire. The mean score (mean), standard error (se), t Welch’s statistics, corresponding p-value, and effect size (Hedge’s g), are reported for each scale. Significant group differences (p < 0.1) are marked in red.

|  | ACTIVE (n=13) | | SHAM (n=12) | |  |  |  |
| --- | --- | --- | --- | --- | --- | --- | --- |
| QUESTIONNAIRES | mean | se | mean | se | t (df=23) | p | Hedges' g |
| FWT | 23.62 | 0.89 | 22.25 | 0.91 | 1.076 | 0.293 | 0.416 |
| PSQI | 5.54 | 0.62 | 7.00 | 1.04 | -1.211 | 0.241 | -0.473 |
| ESS | 8.15 | 1.14 | 7.58 | 0.58 | 0.445 | 0.662 | 0.170 |
| KSS | 3.54 | 0.31 | 3.58 | 0.48 | -0.079 | 0.938 | -0.031 |
| QoL - PHYSICAL_HEALTH | 15.62 | 0.87 | 16.00 | 0.58 | -0.369 | 0.716 | -0.142 |
| QoL - PSYCHOLOGICAL | 13.54 | 0.85 | 13.83 | 0.75 | -0.260 | 0.797 | -0.100 |
| QoL - SOCIAL | 14.23 | 0.68 | 15.17 | 1.12 | -0.714 | 0.484 | -0.279 |
| QoL - ENVIRONMENT | 14.77 | 0.56 | 15.58 | 0.51 | -1.074 | 0.294 | -0.415 |
| SIMPAQ_TOTAL* | 17.34 | 0.95 | 19.58 | 0.46 | -2.135 | 0.047 | -0.816 |
| SIMPAQ_MVPA | 1.55 | 0.30 | 1.99 | 0.27 | -1.086 | 0.289 | -0.419 |
| TMT_A | 26.60 | 3.13 | 26.59 | 2.71 | 0.002 | 0.999 | 0.001 |
| TMT_B | 46.49 | 4.91 | 48.97 | 6.56 | -0.304 | 0.765 | -0.118 |
| TMT (B-A) | 19.89 | 4.84 | 22.38 | 7.19 | -0.288 | 0.776 | -0.112 |
| DEPRESSION | 4.23 | 0.79 | 3.75 | 0.95 | 0.391 | 0.700 | 0.152 |
| ANXIETY | 8.54 | 1.01 | 7.75 | 1.37 | 0.463 | 0.648 | 0.180 |
| ALERTNESS | 3.77 | 0.17 | 3.67 | 0.28 | 0.318 | 0.754 | 0.123 |
| MOTIVATION | 3.92 | 0.24 | 3.92 | 0.15 | 0.022 | 0.982 | 0.009 |
| EXPECTATION | 3.62 | 0.27 | 3.17 | 0.21 | 1.314 | 0.202 | 0.509 |
| SADNESS | 3.39 | 0.29 | 3.75 | 0.31 | -0.870 | 0.393 | -0.337 |
| POSITIVE AFFECT | 32.61 | 2.35 | 30.42 | 2.12 | 0.694 | 0.494 | 0.268 |
| NEGATIVE AFFECT | 14.15 | 0.92 | 11.92 | 0.88 | 1.749 | 0.094 | 0.676 |

# Cognitive state during training

Table S4. Output of a 2-way mixed ANOVA (between subjects: STIMULATION: ACTIVE, CONTROL x within-subject: TIME: T1, T2, T3) on attitude and expectation, significant findings are marked with **.

| ALERTNESS |  | **df** | **F** | **p** | **η²_p_** |
| --- | --- | --- | --- | --- | --- |
|  | SESSION****** | 2,46 | 4.296 | 0.019 | 0.157 |
|  | STIMULATION | 1,23 | 0.308 | 0.584 | 0.013 |
|  | SESSION ✻ STIMULATION | 2,46 | 0.207 | 0.814 | 0.009 |
| MOTIVATION |  | **df** | **F** | **p** | **η²_p_** |
|  | SESSION | 2,46 | 1.105 | 0.340 | 0.046 |
|  | STIMULATION | 1,23 | 1.429 | 0.244 | 0.058 |
|  | SESSION ✻ STIMULATION | 2,46 | 0.754 | 0.476 | 0.032 |
| SADNESS |  | **df** | **F** | **p** | **η²_p_** |
|  | SESSION | 2,46 | 1.578 | 0.217 | 0.064 |
|  | STIMULATION | 1,23 | 1.036 | 0.319 | 0.043 |
|  | SESSION ✻ STIMULATION | 2,46 | 0.658 | 0.523 | 0.028 |
| EXPECTATION |  | **df** | **F** | **p** | **η²_p_** |
|  | SESSION | 2,46 | 0.040 | 0.101 | 0.904 |
|  | STIMULATION | 1,23 | 1.391 | 0.250 | 0.057 |
|  | SESSION ✻ STIMULATION | 2,46 | 0.120 | 0.302 | 0.741 |
| POSITIVE AFFECT |  | **df** | **F** | **p** | **η²_p_** |
|  | SESSION** | 2,46 | 3.838 | 0.029 | 0.143 |
|  | STIMULATION | 1,23 | 0.559 | 0.470 | 0.024 |
|  | SESSION ✻ STIMULATION | 2,46 | 0.768 | 0.462 | 0.032 |
| NEGATIVE AFFECT |  | **df** | **F** | **p** | **η²_p_** |
|  | SESSION | 2,46 | 1.233 | 0.301 | 0.051 |
|  | STIMULATION | 1,23 | 0.845 | 0.367 | 0.035 |
|  | SESSION ✻ STIMULATION | 2,46 | 0.796 | 0.457 | 0.033 |
| KSS |  | **df** | **F** | **p** | **η²_p_** |
|  | SESSION | 2,46 | 2.427 | 0.100 | 0.095 |
|  | STIMULATION | 1,23 | 0.001 | 0.972 | 0.000 |
|  | SESSION ✻ STIMULATION | 2,46 | 1.866 | 0.166 | 0.075 |

# Strategy and task difficulty

We collected feedback on strategy use during the training, asking participants if they used the strategy instructions given to them, or a different one, and, if so, how efficient they feel the strategy was in improving their performance, on a scale from 1 to 10. We found no significant association between STIMULATION groups and those who used the strategy instructions given during the training (X^2^_(1, N=25)_ = 0.962, p = 0.327), or a different strategy (X^2^_(1, N=25)_ = 1.128, p = 0.288). There was also no difference in perceived efficacy between ACTIVE and SHAM. Perceived efficacy correlated significantly (Pearson’s r=0.485, p=0.014, N=25) with training gains.

Table S5. percentage of individual using a strategy for more than 50% of the sessions (excluding follow up)

|  | GROUP | Valid | % | se |
| --- | --- | --- | --- | --- |
| Used strategy given | Active | 13 | 0.69 | 0.13 |
|  | Control | 12 | 0.50 | 0.15 |
| Used ‘a’ strategy | Active | 13 | 1.00 | 0.00 |
|  | Control | 12 | 0.92 | 0.08 |
| Efficacy of the strategy used | Active | 13 | 6.71 | 0.30 |
|  | Control | 12 | 7.17 | 0.34 |


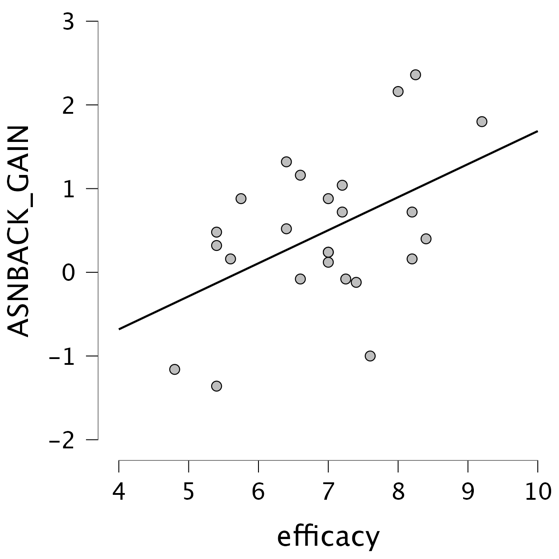


Figure S1 Correlation between perceived strategy efficacy and training-related gains.

# Possible adverse effects of brain stimulation

Possible adverse effects were collected from participants after each stimulation session, together with the likelihood of such effects being caused by stimulation. Table S6 summarizes the number of participants reporting a side effect in at least one of the sessions. There was no difference in participants’ group allocation guesses, indicating that subjects were blind to the stimulation group (Fisher’s exact p = 0.594)

Table S6. Number n of individuals in the ACTIVE and CONTROL groups reporting a side effect in at least one of the training sessions.

| SIDE EFFECT | ACTIVE (n=13) | SHAM (n=13) |
| --- | --- | --- |
| HEADACHE | 4 | 5 |
| PAIN IN NECK | 3 | 2 |
| ITCHING | 10 | 7 |
| SLEEPINESS | 7 | 3 |
| TROUBLE CONCENTRATING | 7 | 9 |
| ACUTE MOOD CHANGE | 0 | 0 |
| FATIGUE | 5 | 1 |
| NAUSEA | 1 | 0 |
| MUSCLE TWITCH IN FACE OR NECK | 3 | 0 |
| TINGLING | 13 | 12 |
| BURNING | 11 | 9 |
| EPILEPTIC SEIZURE | 0 | 0 |
| LIGHT FLASHES | 0 | 0 |
| UNCOMFORTABLE FEELING | 5 | 6 |

# Outcome tasks

## Change detection task

Table S7. Independent t-test of the effect of STIMULATION on changes of dependent variables (ΔRT, ΔD’, ΔC, and ΔK) with respect to baseline in the CD task.

| ΔRT | | | | | | |
| --- | --- | --- | --- | --- | --- | --- |
|  | ACTIVE | SHAM | df | t | p | H_g_ |
| T4 | -232 ± 39 | -157 ± 36 | 23 | 1.404 | 0.174 | 0.544 |
| T5 | -160 ± 50 | -205 ± 34 | 21 | 0.230 | 0.820 | 0.093 |
| ΔD' | | | | | | |
|  | ACTIVE | SHAM | df | t | p | H_g_ |
| T4 | 0.20 ± 0.06 | 0.05 ± 0.10 | 23 | 1.319 | 0.200 | 0.511 |
| T5 | 0.11 ± 0.09 | 0.13 ± 0.11 | 21 | 0.183 | 0.856 | 0.074 |
| ΔC | | | | | | |
|  | ACTIVE | SHAM | df | t | p | H_g_ |
| T4 | -0.30 ± 0.05 | -0.12 ± 0.06 | 23 | 2.310 | 0.030 | 0.894 |
| T5 | -0.34 ± 0.06 | -0.36 ± 0.05 | 21 | 0.687 | 0.500 | 0.278 |
| ΔK | | | | | | |
|  | ACTIVE | SHAM | df | t | p | H_g_ |
| T4 | -0.09 ± 0.10 | -0.04 ± 0.15 | 23 | 0.306 | 0.762 | 0.119 |
| T5 | -0.26 ± 0.13 | -0.13 ± 0.24 | 21 | 0.516 | 0.612 | 0.209 |

Table S8. T-test of changes in each stimulation group against zero in the CD task.

| POST-TEST T4 | | | | |
| --- | --- | --- | --- | --- |
|  | ACTIVE > 0 | | SHAM > 0 | |
|  | t(df=12) | p | t(df=11) | p |
| ΔRT | -5.907 | < .001 | -4.415 | 0.001 |
| ΔD' | 3.210 | 0.007 | 0.491 | 0.633 |
| ΔC | -5.769 | < .001 | -2.182 | 0.052 |
| ΔK | -0.898 | 0.387 | -0.244 | 0.812 |
| FOLLOW UP T5 | | | | |
|  | ACTIVE > 0 | | SHAM > 0 | |
|  | t(df=12) | p | t(df=9) | p |
| ΔRT | -3.186 | 0.008 | -5.974 | < .001 |
| ΔD' | 1.173 | 0.264 | 1.145 | 0.282 |
| ΔC | -5.491 | < .001 | -6.863 | < .001 |
| ΔK | -2.009 | 0.068 | -0.520 | 0.616 |

## SNBACK task

Table S9. Independent t-test of the effect of STIMULATION on changes of dependent variables (ΔRT, ΔD’ and ΔC) with respect to baseline in the SNBACK task.

| ΔRT | | | | | | |
| --- | --- | --- | --- | --- | --- | --- |
|  | ACTIVE | SHAM | df | t | p | H_g_ |
| T4 | -203 ± 27 | -166 ± 28 | 23 | 0.945 | 0.354 | 0.366 |
| T5 | -155 ± 31 | -136 ± 36 | 21 | 0.402 | 0.692 | 0.163 |
| ΔD’ | | | | | | |
|  | ACTIVE | SHAM | df | t | p | H_g_ |
| T4 | 0.73 ± 0.20 | 0.87 ± 0.21 | 23 | 0.503 | 0.620 | 0.195 |
| T5 | 0.71 ± 0.21 | 0.89 ± 0.24 | 21 | 0.575 | 0.572 | 0.233 |
| ΔC | | | | | | |
|  | ACTIVE | SHAM | df | t | p | H_g_ |
| T4 | 0.13 ± 0.05 | 0.05 ± 0.05 | 23 | 0.968 | 0.340 | 0.375 |
| T5 | 0.19 ± 0.07 | 0.06 ± 0.08 | 21 | 1.130 | 0.271 | 0.458 |

Table S10. T-test of changes in each stimulation group against zero in the SNBACK task.

| POST-TEST T4 | | | | |
| --- | --- | --- | --- | --- |
|  | ACTIVE > 0 | | SHAM > 0 | |
|  | t(df=12) | p | t(df=11) | p |
| ΔRT | -6.855 | < .001 | -6.572 | < .001 |
| ΔD' | 4.044 | 0.002 | 3.742 | 0.003 |
| ΔC | 2.329 | 0.038 | 0.908 | 0.383 |
| FOLLOW UP T5 | | | | |
|  | ACTIVE > 0 | | SHAM > 0 | |
|  | t(df=12) | p | t(df=9) | p |
| ΔRT | -5.586 | < .001 | -3.338 | 0.009 |
| ΔD' | 4.953 | < .001 | 2.888 | 0.018 |
| ΔC | 2.511 | 0.027 | 0.799 | 0.445 |

Table S11 Comparison of short-term changes in amplitude (ΔA_ST_) and modulation (ΔM_ST_) for each component between ACTIVE and SHAM group.

| **ΔA_ST_ (averaged over ‘n’)** | | | | | | | | |
| --- | --- | --- | --- | --- | --- | --- | --- | --- |
|  |  | ACTIVE | | SHAM | |  |  |  |
|  | n | mu | se | mu | se | F(1,23) | p | $\eta_{p}^{2}$ |
| P1 | 25 | -0.43 | 0.22 | -0.26 | 0.23 | 0.280 | 0.602 | 0.012 |
| N1 | 25 | -0.74 | 0.27 | -0.82 | 0.28 | 0.041 | 0.841 | 0.002 |
| P2 | 25 | -0.51 | 0.46 | -0.69 | 0.48 | 0.075 | 0.787 | 0.003 |
| N2 | 25 | 0.96 | 0.71 | 1.01 | 0.74 | 0.002 | 0.966 | <0.001 |
| P3 | 25 | 1.92 | 0.80 | 1.64 | 0.84 | 0.058 | 0.812 | 0.002 |
| SW | 25 | 0.55 | 0.40 | 0.24 | 0.42 | 0.280 | 0.602 | 0.012 |
| **ΔM_ST_ (averaged over ‘n’)** | | | | | | | | |
|  |  | ACTIVE | | SHAM | |  |  |  |
|  | n | mu | se | mu | se | F(1,23) | p | $\eta_{p}^{2}$ |
| P1 | 25 | 0.12 | 0.10 | 0.20 | 0.11 | 0.278 | 0.603 | 0.012 |
| N1 | 25 | 0.09 | 0.17 | 0.22 | 0.18 | 0.270 | 0.308 | 0.012 |
| P2 | 25 | 0.11 | 0.21 | -0.27 | 0.22 | 1.570 | 0.223 | 0.064 |
| N2 | 25 | 0.61 | 0.28 | -0.04 | 0.30 | 2.453 | 0.131 | 0.096 |
| P3 | 25 | 0.50 | 0.30 | -0.03 | 0.31 | 1.457 | 0.240 | 0.060 |
| SW | 25 | 0.05 | 0.17 | -0.08 | 0.18 | 0.281 | 0.601 | 0.012 |

Table S12 Comparison of short-term changes in amplitude (ΔA_LT_) and modulation (ΔM_LT_) for each component between ACTIVE and SHAM group.

| **ΔA_LT_ (averaged over 'n')** | | | | | | | | |
| --- | --- | --- | --- | --- | --- | --- | --- | --- |
|  |  | ACTIVE | | SHAM | |  |  |  |
|  | n | mu | se | mu | se | F(1,21) | p | $\eta_{p}^{2}$ |
| P1 | 23 | -0.15 | 0.15 | -0.38 | 0.17 | 1.017 | 0.325 | 0.046 |
| N1 | 23 | -0.36 | 0.18 | -0.55 | 0.21 | 0.513 | 0.482 | 0.024 |
| P2 | 23 | -1.36 | 0.52 | -1.80 | 0.60 | 0.313 | 0.582 | 0.015 |
| N2 | 23 | 0.11 | 0.64 | -0.72 | 0.73 | 0.734 | 0.401 | 0.034 |
| P3 | 23 | 0.83 | 0.76 | -0.23 | 0.86 | 0.847 | 0.368 | 0.039 |
| SW | 23 | -0.24 | 0.32 | -0.95 | 0.36 | 2.145 | 0.158 | 0.093 |
| **ΔM_LT_ (averaged over ‘n’)** | | | | | | | | |
|  |  | ACTIVE | | SHAM | |  |  |  |
|  | n | mu | se | mu | se | F(1,21) | p | $\eta_{p}^{2}$ |
| P1 | 23 | 0.47 | 0.22 | 0.58 | 0.26 | 0.102 | 0.752 | 0.005 |
| N1 | 23 | 0.38 | 0.20 | 0.47 | 0.23 | 0.080 | 0.781 | 0.004 |
| P2 | 23 | 0.44 | 0.21 | 0.27 | 0.24 | 0.291 | 0.595 | 0.014 |
| N2 | 23 | 0.19 | 0.38 | 0.65 | 0.43 | 0.619 | 0.440 | 0.029 |
| P3 | 23 | 0.36 | 0.48 | 0.47 | 0.55 | 0.025 | 0.876 | 0.001 |
| SW | 23 | 0.07 | 0.20 | 0.54 | 0.22 | 2.559 | 0.125 | 0.109 |

Table S13. Outcome of a one-sample t-test against zero for short-term and long-term changes in amplitude and modulation for each component.

|  |  | ACTIVE | | | SHAM | | |
| --- | --- | --- | --- | --- | --- | --- | --- |
| P1 |  | **t** | **df** | **p** | **t** | **df** | **p** |
|  | $\Delta A_{ST}$ | -1.858 | 12 | 0.088 | -1.166 | 11 | 0.268 |
|  | $\Delta A_{LT}$ | -1.149 | 12 | 0.273 | -1.846 | 9 | 0.098 |
|  | $\Delta M_{ST}$ | 1.179 | 12 | 0.261 | 1.744 | 11 | 0.109 |
|  | $\Delta M_{LT}$ | 2.818 | 12 | 0.016 | 1.796 | 9 | 0.106 |
| N1 |  | **t** | **df** | **p** | **t** | **df** | **p** |
|  | $\Delta A_{ST}$ | -3.706 | 12 | 0.003 | -2.475 | 11 | 0.031 |
|  | $\Delta A_{LT}$ | -2.501 | 12 | 0.028 | -2.187 | 9 | 0.057 |
|  | $\Delta M_{ST}$ | 0.702 | 12 | 0.496 | 1.008 | 11 | 0.335 |
|  | $\Delta M_{LT}$ | 1.709 | 12 | 0.113 | 2.417 | 9 | 0.039 |
| P2 |  | **t** | **df** | **p** | **t** | **df** | **p** |
|  | $\Delta A_{ST}$ | -1.083 | 12 | 0.3 | -1.49 | 11 | 0.164 |
|  | $\Delta A_{LT}$ | -2.224 | 12 | 0.046 | -4.236 | 9 | 0.002 |
|  | $\Delta M_{ST}$ | 0.533 | 12 | 0.604 | -1.205 | 11 | 0.253 |
|  | $\Delta M_{LT}$ | 2.085 | 12 | 0.059 | 1.118 | 9 | 0.293 |
| N2 |  | **t** | **df** | **p** | **t** | **df** | **p** |
|  | $\Delta A_{ST}$ | 1.606 | 12 | 0.134 | 1.189 | 11 | 0.259 |
|  | $\Delta A_{LT}$ | 0.178 | 12 | 0.862 | -0.927 | 9 | 0.378 |
|  | $\Delta M_{ST}$ | 1.877 | 12 | 0.085 | -0.143 | 11 | 0.889 |
|  | $\Delta M_{LT}$ | 0.488 | 12 | 0.634 | 1.604 | 9 | 0.143 |
| P3 |  | **t** | **df** | **p** | **t** | **df** | **p** |
|  | $\Delta A_{ST}$ | 3.255 | 12 | 0.007 | 1.61 | 11 | 0.136 |
|  | $\Delta A_{LT}$ | 1.317 | 12 | 0.212 | -0.222 | 9 | 0.83 |
|  | $\Delta M_{ST}$ | 1.577 | 12 | 0.141 | -0.083 | 11 | 0.935 |
|  | $\Delta M_{LT}$ | 1.057 | 12 | 0.311 | 0.667 | 9 | 0.521 |
| SW |  | **t** | **df** | **p** | **t** | **df** | **p** |
|  | $\Delta A_{ST}$ | 1.349 | 12 | 0.202 | 0.584 | 11 | 0.571 |
|  | $\Delta A_{LT}$ | -0.734 | 12 | 0.477 | -2.756 | 9 | 0.022 |
|  | $\Delta M_{ST}$ | 0.28 | 12 | 0.784 | -0.473 | 11 | 0.645 |
|  | $\Delta M_{LT}$ | 0.339 | 12 | 0.697 | 2.143 | 9 | 0.061 |

Table S15 Average number of removed components or interpolated electrodes across sessions and n level, for each individual. The mean percentage of rejected trials across sessions and level is reported.

|  | REJECTED TRIALS  (%) | | INTERPOLATED ELECTRODES (n) | | REMOVED ICs (n) | |
| --- | --- | --- | --- | --- | --- | --- |
|  | mean | sd | mean | sd | mean | sd |
| OVERALL | 6.3 | 9.7 | 0.6 | 1.2 | 8.3 | 4.0 |
| sub01 | 4.0 | 3.2 | 1.7 | 1.5 | 4.7 | 1.2 |
| sub02 | 1.2 | 1.9 | 0.0 | 0.0 | 7.8 | 2.0 |
| sub03 | 4.4 | 2.8 | 0.9 | 0.8 | 4.7 | 2.0 |
| sub04 | 2.4 | 1.2 | 0.0 | 0.0 | 8.2 | 2.9 |
| sub05 | 2.8 | 2.5 | 0.0 | 0.0 | 14.3 | 1.6 |
| sub06 | 8.9 | 6.1 | 4.0 | 3.0 | 16.1 | 3.5 |
| sub07 | 15.9 | 4.6 | 0.3 | 0.7 | 5.8 | 2.0 |
| sub08 | 31.4 | 21.4 | 1.7 | 0.9 | 8.8 | 2.9 |
| sub09 | 7.9 | 10.8 | 0.4 | 0.5 | 10.2 | 4.7 |
| sub10 | 1.4 | 1.0 | 1.6 | 1.1 | 9.9 | 2.9 |
| sub11 | 5.2 | 3.4 | 1.0 | 0.7 | 7.9 | 1.8 |
| sub12 | 3.2 | 2.5 | 1.4 | 1.1 | 10.0 | 3.9 |
| sub13 | 5.5 | 4.0 | 0.3 | 0.7 | 6.7 | 2.0 |
| sub14 | 7.2 | 6.5 | 0.0 | 0.0 | 7.3 | 3.3 |
| sub15 | 1.1 | 1.7 | 0.1 | 0.3 | 11.3 | 3.1 |
| sub16 | 0.7 | 0.6 | 0.2 | 0.4 | 10.2 | 4.2 |
| sub17 | 2.7 | 1.5 | 0.2 | 0.4 | 5.2 | 1.5 |
| sub18 | 9.2 | 8.3 | 0.1 | 0.3 | 6.9 | 2.8 |
| sub19 | 4.0 | 1.9 | 0.0 | 0.0 | 4.7 | 1.9 |
| sub20 | 1.4 | 1.0 | 0.3 | 0.5 | 7.0 | 2.0 |
| sub21 | 5.8 | 2.7 | 0.0 | 0.0 | 10.3 | 2.1 |
| sub22 | 14.9 | 14.2 | 0.2 | 0.4 | 7.3 | 3.3 |
| sub23 | 8.7 | 20.5 | 0.3 | 0.7 | 8.7 | 2.6 |
| sub24 | 7.5 | 5.2 | 0.3 | 0.5 | 4.3 | 1.5 |
| sub25 | 2.5 | 5.4 | 0.0 | 0.0 | 5.6 | 2.5 |
| sub26 | 3.4 | 2.5 | 0.9 | 1.1 | 12.8 | 3.3 |
